# Awareness of Iranian Medical Sciences Students Towards Basic Life Support; a Cross-Sectional study

**DOI:** 10.22037/aaem.v9i1.1231

**Published:** 2021-05-20

**Authors:** Meisam Akhlaghdoust, Saeid Safari, Poorya Davoodi, Shaghayegh Soleimani, Maryam Khorasani, Fatemeh Raoufizadeh, Hosna Karimi, Elahe Etesami, Zeynab Hamzehloei, Seyedeh Sara Sadeghi, Ladan Heidaresfahani, Tooba Ebadi Fard Azar, Haniyeh Afshari Badrloo

**Affiliations:** 1 Functional Neurosurgery Research Center, Shahid Beheshti University of Medical Sciences, Tehran, Iran.; 2 USERN Office, Functional Neurosurgery Research Center, Shahid Beheshti University of Medical Sciences, Tehran, Iran.; 3 Pars Advanced and Minimally Invasive Medical Manners Research Center, Pars Hospital, Iran University of Medical Sciences, Tehran, Iran.; 4 Islamic Azad University, TehranMedical Sciences Branch, Tehran, Iranslamic Azad University, TehranMedical Sciences Branch, Tehran, Iran.; 5 Science and Research Branch, Islamic Azad University, Tehran, Iran.

**Keywords:** Awareness, cardiopulmonary resuscitation, heart arrest, attitude

## Abstract

**Introduction:**

Augmentation of the number of trained basic life support (BLS) providers can remarkably reduce the number of cardiac arrest victims. The aim of this study was to evaluate the level of BLS awareness among students of medical sciences in Iran.

**Methods:**

This multicenter cross-sectional study was performed on medical students at the 4 major medical schools in Tehran, the capital of Iran, between Jan 2018 and Feb 2019, using convenience sampling method. The level of medical sciences students’ awareness of BLS was measured using an international questionnaire.

**Results:**

Finally, 1210 students with the mean age of 21.2 ± 2.3 years completed the survey (79% female). 133 (10.9%) students had CPR experience and none had received any formal training. None of the responders could answer all questions correctly. The mean awareness score of participants was 11.93 ± 2.87 (range: 10.13 -17.25). The awareness score of participants was high in 49 (4.04 %) participants, moderate in 218 (18.01%), and low in 943 (77.93%) of studied cases.

**Conclusion:**

Based on the findings of this study, more than 70% of the studied medical sciences students obtained a low score on BLS awareness.

## 1. Introduction: 

Cardiac arrest is a fatal condition responsible for a large number of deaths in the modern world, and it has remained common worldwide (1–3). Deaths caused by cardiac arrest can be prevented via simple maneuvers and skills most of the time (5). Cardio-pulmonary resuscitation (CPR) is a life-saving and valuable technique that was invented back in 1960 (6); it is indeed a facile procedure that permits almost everyone to sustain life and decreases mortality up to 50% in golden minutes after cardiac and respiratory arrests (7,8).

Based on the place in which a cardiac arrest takes place, it is divided into two categories of out-of-hospital cardiac arrest (OHCA) and in-hospital cardiac arrest (IHCA). OHCA occurs approximately in 19-104 per 100,000 persons each year (0.019-0.104%), and 10% of them are said to be saved at the hospital (9). Statics present that 350,000 people in Europe die annually because of OHCA (9). In the USA, OHCA is responsible for 760,000 deaths per year (10). 

Augmentation of the number of trained basic life support (BLS) providers can remarkably reduce the number of cardiac arrest victims (16). Therefore, many countries worldwide have integrated these topics into the curricula of their educational centers or even workplaces believing that any individual in the society should have sufficient knowledge and awareness to provide BLS when needed (18). Having this in mind, expectation from physicians and paramedical staff is naturally higher as their career requires this knowledge. 

This study aimed to evaluate the level of BLS awareness among Iranian medical sciences students studying in four major Iranian medical schools.

## 2. Methods:

***2.1. Study design and participants***

This multicenter cross-sectional study was performed on medical students studying in the 4 major medical schools (Tehran Medical Sciences of Islamic Azad University, Tehran University of Medical Sciences, Shahid Beheshti University of Medical Sciences, and Iran University of Medical Sciences) in Tehran, the capital of Iran, between Jan 2018 and Feb 2019, using convenience sampling method. The level of medical sciences students’ awareness of BLS was measured using an international questionnaire. The study protocol was approved by Ethics committee of Iran University of Medical Sciences (IR.IUMS.REC.1399.1291). 

***2.2. Participants***

Being a student in one of the fields of medical sciences (medicine, nursing, and midwifery) was the inclusion criterion. There was not any sex or age limitation in this study. Incomplete questionnaires were excluded (29 questionnaire). 

***2.3. Data gathering***

Demographic data, age, CPR experience, sex, educational status, and attendance of BLS courses were collected using a checklist. In addition, an international questionnaire that measures the level of awareness about BLS was used for evaluating the awareness level of participants regarding BLS. 

We used the Persian version of the international questionnaire measuring the awareness of participants regarding BLS. This questionnaire was designed by Özbilgin Ş et.al. based on the latest version of AHA guideline. The international questionnaire measuring awareness of BLS has 20 multiple-choice questions and each question has 1 point (19). The range of scores of this questionnaire is 16-20 (high), 11-15 (moderate), and 0-10 (Low). Persian version of this questionnaire is validated by Ziabari et al. 2019 (6). 

***2.4. Statistical analysis***

 A required sample size of 1210 participants, was calculated using Rao soft software. For statistical analysis, SPSS software version 22 (SPSS Inc. Chicago, IL, United States) was used. The findings were presented as mean ± standard deviation or frequency and percentage.

## 3. Results:

Finally, 1210 students with the mean age of 21.2 ± 2.3 years completed the survey (79% female). Baseline characteristics of studied participants are presented in table 1. Among the participants, 133 (10.9%) had CPR experience and none had received any formal training. Table 2 shows the results of students’ responses to 20 questions regarding BLS awareness. None of the responders could answer all questions correctly. The mean awareness score of participants was 11.93 ± 2.87 (range: 10.13 -17.25). The awareness score of participants was high in 49 (4.04 %) participants, moderate in 218 (18.01%), and low in 943 (77.93%) of studied cases.

## 4. Discussion: 

Based on the findings of this study, more than 70% of the studied medical sciences students obtained a low score on BLS awareness. 

Nowadays, with the growth of cardio-pulmonary diseases, the rate of cardiac arrest has been remarkably increasing (3-6). It is expected from a majority of community members to be efficiently aware of BLS (especially CPR) (7). CPR is one of the essential skills that people of a society have to learn because it is a life-saving skill and can reduce the number of OHCA victims (8). Based on the reports, performing proper basic life support can reduce the mortality rate, especially in OHCA (3-5, 7, 9).

The Findings in this cross-sectional study showed low awareness of BLS among medical sciences students. About 90% of cases had no experience of CPR, and more than 95% of participants had no idea about safety, and they did not know what they have to do when someone lies unresponsive on the street. Many people do not like to give mouth-to-mouth CPR due to health issues. According to the guideline of the American heart association, if someone does not like to give mouth-to-mouth or is not able to do it, that person should carry out Hands-Only CPR (13). The results demonstrated that only 11.3% of participants knew it. Based on these results, medical students' awareness of BLS is very low. Samiha Jarrah et al. had done research evaluating awareness and knowledge of BLS, and the results were similar to our study (3). They explained that the rate of knowledge and awareness of BLS is low. 

In another study, Neha Baduni et al. did a research on the knowledge and awareness of BLS among dental practitioners (20). 104 persons had participated in their study, and results showed that none of them had complete awareness regarding BLS. Singh et al. conducted a survey among 241 dentists (16). Approximately 53% of them had not participated in any course about CPR or BLS.

In our study, none of the responders could answer all questions correctly, and none had received any formal training. The awareness of BSL in infants and children was low among participants. Only 7% of participants knew the correct depth of compression during CPR in children and 6.4% in neonates. 

Since medical students make up the future of the country's healthcare system, the results of this study should be taken seriously. This study has shown a need for supplementary BLS training in curricula of Iran’s medical universities. More comprehensive studies could be performed in the future by collaborating with other universities in Iran to evaluate the awareness of medical sciences students regarding BLS. We suggest some new methods in medical students’ programs for teaching BLS including: VR-based serious games, teaching BLS to others, and reinforcing the students’ knowledge and awareness every year.

**Table 1:** Baseline characteristics of studied participants

**Variable **	**Total **
**Age (year)**	
Mean ± SD	21.2 ± 2.3
**Sex **	
Female	956 (79.1)
Male	254 (20.9)
**Educational status **	
Medical student	643 (53.1)
Nursing student	296 (24.5)
Midwifery student	271 (22.4)
**Experience of CPR **	
Yes	133 (11.0)
No	1077 (89.0)
**Attendance of BLS courses **	
Yes	194 (16.1)
No	1016 (83.9)

Data are presented as frequency (%). SD: standard deviation; BLS: basic life support; CPR: cardiopulmonary resuscitation.

**Table 2:** Answers to basic life support awareness questions

**Question**	**Answer**	**Correct **
1. What is the abbreviation of “BLS”?	Basic Life Support	280 (23.1)
2. When you find someone unresponsive on the road, what will your first response be? (Note: You are alone there)	Look for Safety	149 (12.3)
3. If you confirm somebody is not responding to you even after shaking and shouting at him, what will be your immediate action?	Activate EMS	436 (36.0)
4. What is the location for chest compression?	Mid Chest	331 (27.4)
5. What is the location for chest compression in infants?	One fingerbreadth above the nipple line	230 (19.0)
6. If you do not want to give mouth-to-mouth CPR, the following can be done *EXCEPT*	No CPR	137 (11.3)
7. How do you give rescue breathing in infants?	Mouth-to-mouth and nose	364 (30.0)
8. Depth of compression in adults during CPR	1 to 1/2 inches	137 (11.3)
9. Depth of compression in Children during CPR	1/2 to 1/3 depth of chest	85 (7.0)
10. Depth of compression in neonates during CPR	1/2 to 1/3 depth of chest	77 (6.4)
11. Rate of chest compression in adults and Children during CPR	100/min	184 (15.2)
12. Ratio of CPR is (single rescuer in the adult)	30:2	179 (14.8)
13. In a newborn, the chest compression and ventilation ratio is	3:1	79 (6.5)
14. What does abbreviation AED stands for?	Automated External Defibrillator	61 (5.0)
15. What does abbreviation EMS stands for?	Emergency Medical Service	188 (15.5)
16. If you and your friend are having food in a canteen and suddenly your friend starts expressing symptoms of choking, what will be your first response?	Confirm foreign body aspiration by talking to him	401 (33.1)
17. You are witnessing an infant who suddenly started choking while he was playing with the toy, you have confirmed that he is unable to cry (or) cough, what will be your first response?	Back blows and chest compression of five cycles each, then open the mouth and remove foreign body only when it is seen	210 (17.4)
18. You are witnessing an adult unresponsive victim who has been submerged in fresh water and just removed from it. He has spontaneous breathing, but he is unresponsive. What is the first step?	Keep him in recovery position	85 (7.0)
19. You noticed that your colleague has suddenly developed slurring of speech and weakness of right upper limb. Which one of the following can be done?	Possibly stroke, he may require thrombolysis, and hence activate emergency medical services	100 (8.3)
20. You notice a 50-year-old gentleman with retrosternal chest discomfort, profuse sweating, and vomiting. What is next?	Probably myocardial infarction, hence activate EMS, give him an aspirin tablet, and allow him to rest	436 (36.0)

## 5. Limitations

Our study has some limitations. One of them is that it was conducted only in the four Medical Universities in Tehran and did not involve the whole country. This study only demonstrates and evaluates the awareness, and the knowledge and practice regarding BLS was not evaluated in this study. The number of students who completed the survey is not enough to generalize the results to other medical schools in Iran.

## 6. Conclusion:

Based on the findings of this study, more than 70% of the studied medical sciences students obtained a low score on BLS awareness. 

## 7. Declarations:

### 7.1. Acknowledgments

None. 

### 7.2. Author contribution

All authors made substantial contributions, revised the manuscript, and approved the final version for publication. 

### 7.3. Funding sources

The author(s) received no financial support for the research, authorship, and/or publication of this article.

### 7.4. Conflict of interest

None to Declare. 

## References

[1] Ghanem E, Elgazar M, Oweda K, Tarek H, Assaf F, El-Husseny MWA (2018). Awareness of basic life support among Egyptian medical students; a cross-sectional study. Emergency.

[2] Saquib SA, Al-Harthi HM, Khoshhal AA, Shaher AA, Al-Shammari AB, Khan A (2019). Knowledge and attitude about basic life support and emergency medical services amongst healthcare interns in university hospitals: a cross-sectional study. Emerg Med Int.

[3] Jarrah S, Judeh M, AbuRuz ME (2018). Evaluation of public awareness, knowledge and attitudes towards basic life support: a cross-sectional study. BMC Emerg Med.

[4] Zaheer H, Haque Z (2009). Students’ Corner-Awareness about BLS (CPR) among medical students: Status and requirements. J Pak Med Assoc.

[5] Vural M, Koşar MF, Kerimoğlu O, Kızkapan F, Kahyaoğlu S, Tuğrul S (2017). Cardiopulmonary resuscitation knowledge among nursing students: a questionnaire study. Anatol J Cardiol.

[6] Ziabari SMZ, Kasmaei VM, Khoshgozaran L, Shakiba M (2019). Continuous education of basic life support (BLS) through social media; a quasi-experimental study. Arch Acad Emerg Med.

[7] Alanazi A, Bin-Hotan AlM, ALhalyabah H, Alanazi A, Al-oraibi S (2013). Community awareness about cardiopulmonary resuscitation among secondary school students in Riyadh. World J Med Sci.

[8] Gravesteijn BY, Schluep M, Voormolen DC, van der Burgh AC, Miranda DDR, Hoeks SE (2019). Cost-effectiveness of extracorporeal cardiopulmonary resuscitation after in-hospital cardiac arrest: A Markov decision model. Resuscitation.

[9] Duff JP, Topjian A, Berg MD, Chan M, Haskell SE, Joyner Jr BL (2018). 2018 American Heart Association focused update on pediatric advanced life support: an update to the American Heart Association guidelines for cardiopulmonary resuscitation and emergency cardiovascular care. Circulation.

[10] Alimohammadzadeh K, Akhlaghdoust M, Bahrainian SA, Mirzaei A (2017). Survey on Mental Health of Iranian Medical Students: A Cross-sectional Study in Islamic Azad University. Shiraz E-Medical J.

[11] Sangamesh NC, Vidya KC, Pathi J, Singh A (2017). Awareness, attitude, and knowledge of basic life support among medical, dental, and nursing faculties and students in the university hospital. J Int Soc Prev Community Dent.

[12] Al-Shamiri HM, Al-Maweri SA, Shugaa-Addin B, Alaizari NA, Hunaish A (2017). Awareness of basic life support among Saudi dental students and interns. Eur J Dent.

[13] Powers WJ, Rabinstein AA, Ackerson T, Adeoye OM, Bambakidis NC, Becker K (2019). Guidelines for the early management of patients with acute ischemic stroke: 2019 update to the 2018 guidelines for the early management of acute ischemic stroke: a guideline for healthcare professionals from the American Heart Association/American Stroke. Stroke.

[14] Cheng A, Nadkarni VM, Mancini MB, Hunt EA, Sinz EH, Merchant RM (2018). Resuscitation education science: educational strategies to improve outcomes from cardiac arrest: a scientific statement from the American Heart Association. Circulation.

[15] Krammel M, Schnaubelt S, Weidenauer D, Winnisch M, Steininger M, Eichelter J (2018). Gender and age-specific aspects of awareness and knowledge in basic life support. PLoS One.

[16] Zaheer H, Haque Z (2009). Awareness about BLS (CPR) among medical students: Status and requirements. J Pak Med Assoc.

[17] Al-Turki YA, Al-Fraih YS, Jalaly JB, Al-Maghlouth IA, Al-Rashoudi FH, Al-Otaibi AF (2008). Knowledge and attitudes towards cardiopulmonary resuscitation among university students in Riyadh, Saudi Arabia. Saudi med J.

[18] Alharbi MM, Horaib YF, Almutairi OM, Alsuaidan BH, Alghoraibi MS, Alhadeedi FH (2016). Exploring the extent of knowledge of CPR skills among school teachers in Riyadh, KSA. J Taibah Univ Med Sci.

[19] Özbilgin Ş, Akan M, Hancı V, Aygün C, Kuvaki B (2015). Evaluation of public awareness, knowledge and attitudes about cardiopulmonary resuscitation: Report of İzmir. Turk Anesteziyoloji ve Reanimasyon Dern Derg.

[20] Baduni N, Prakash P, Srivastava D, Sanwal M, Singh B (2014 ). Awareness of basic life support among dental practitioners. Natl J Maxillofac Surg [Internet].

[21] Chen M, Wang Y, Li X, Hou L, Wang Y, Liu J (2017). Public knowledge and attitudes towards bystander cardiopulmonary resuscitation in China. Biomed Res Int.

[22] Abbas A, Bukhari SI, Ahmad F (2011). Knowledge of first aid and basic life support amongst medical students: a comparison between trained and un-trained students. J Pak Med Assoc.

[23] Irfan B, Zahid I, Khan MS, Khan OAA, Zaidi S, Awan S (2019). Current state of knowledge of basic life support in health professionals of the largest city in Pakistan: a cross-sectional study. BMC Health Serv Res.

